# Regulated Cell Death in Prostate Cancer: Immunometabolic Crosstalk, Therapeutic Resistance, and Biomarker-Guided Combination Strategies

**DOI:** 10.3390/cancers18122014

**Published:** 2026-06-22

**Authors:** Chunlin Wang, Ning Li

**Affiliations:** Department of Urology, The Fourth Affiliated Hospital of China Medical University, Shenyang 110032, China; wangchunlin@cmu.edu.cn

**Keywords:** prostate cancer, regulated cell death, ferroptosis, autophagy, tumor microenvironment, immunogenic cell death

## Abstract

Prostate cancer remains a major therapeutic challenge, particularly after progression to castration-resistant disease, where persistent androgen receptor signaling, metabolic adaptation, immune escape, and treatment resistance jointly limit clinical benefit. Regulated cell death (RCD) is increasingly recognized not only as an endpoint of tumor cell elimination but also as a dynamic regulator of prostate cancer progression, therapeutic vulnerability, and tumor–immune interactions. In this review, we propose an immunometabolic framework in which androgen receptor signaling, lipid and redox metabolic reprogramming, oxidative stress, and therapeutic pressure converge to shape the susceptibility of prostate cancer cells to distinct RCD modalities. We focus on autophagy and ferroptosis as two extensively studied and translationally relevant pathways, while also discussing emerging roles of necroptosis, pyroptosis, and cuproptosis. Particular attention is given to how RCD-associated signals, including damage-associated molecular patterns, inflammatory mediators, and lipid peroxidation products, may remodel the tumor immune microenvironment and influence the transition between immune-cold and immune-inflamed phenotypes. We further summarize RCD-targeted therapeutic strategies, including ferroptosis induction, autophagy inhibition, nanodrug delivery systems, rational combination therapy, and biomarker-guided patient stratification. Finally, we discuss key translational barriers, including context-dependent biological effects, limited clinical validation, tumor heterogeneity, adaptive resistance, and insufficient predictive biomarkers. By integrating cell death biology with metabolic reprogramming, immune remodeling, and therapeutic resistance, this review highlights RCD as a promising but context-dependent therapeutic vulnerability in advanced prostate cancer.

## Simple Summary

Prostate cancer is one of the most common cancers in men, and advanced disease can become difficult to treat when tumors no longer respond well to standard hormone-based therapies. One reason for treatment failure is that cancer cells learn to avoid the normal processes that should lead damaged or dangerous cells to die. This review explains how different forms of regulated cell death are involved in prostate cancer growth, treatment resistance, and interactions with the immune system. We also discuss how these cell death pathways are connected with cancer metabolism and how they may be used to design new treatment combinations, drug delivery approaches, and biomarker-based strategies. By summarizing current knowledge and remaining challenges, this review may help researchers identify more effective ways to target advanced prostate cancer and guide future studies toward more personalized treatment.

## 1. Introduction

Prostate cancer (PCa) is the second most frequently diagnosed malignancy among men worldwide and remains a major cause of cancer-related mortality [1]. Its incidence continues to rise with population aging in many regions worldwide [1]. Although localized disease can often be effectively managed by surgery or radiotherapy, advanced and metastatic prostate cancer remains associated with unfavorable clinical outcomes [2].

Androgen receptor (AR) signaling plays a central role in prostate cancer development and progression [3,4]. Most early-stage tumors are androgen-dependent and initially respond well to androgen deprivation therapy (ADT). However, nearly all patients eventually progress to castration-resistant prostate cancer (CRPC), a lethal stage characterized by persistent AR signaling despite androgen depletion [5,6]. Multiple mechanisms contribute to CRPC, including *AR* amplification, *AR* mutation, expression of constitutively active AR splice variants such as AR-V7, and activation of alternative survival pathways, particularly phosphoinositide 3-kinase/protein kinase B (PI3K/AKT) signaling [7,8,9].

Beyond genetic alterations, metabolic reprogramming has emerged as another hallmark of prostate cancer progression [10,11]. By reshaping lipid, amino acid, and redox metabolism, prostate cancer cells acquire enhanced survival capacity under therapeutic and microenvironmental stress [10,11]. These metabolic adaptations not only support tumor growth but also profoundly influence the susceptibility of cancer cells to distinct forms of regulated cell death (RCD) [12,13].

RCD comprises a group of tightly regulated biological processes that determine cellular fate in response to physiological and pathological stimuli [14]. Unlike accidental necrosis, RCD is orchestrated by specific molecular signaling networks [14]. In addition to classical and well-characterized processes such as apoptosis and autophagy-associated cell death, recently characterized modalities including ferroptosis, necroptosis, pyroptosis, and cuproptosis have greatly expanded the landscape of RCD [14,15].

At the basic mechanistic level, RCD can involve both caspase-dependent and caspase-independent pathways [14]. Caspase-dependent apoptosis is mainly mediated through the intrinsic mitochondrial pathway and the extrinsic death receptor pathway, both of which converge on executioner caspases that coordinate apoptotic cell dismantling [14,16]. In contrast, caspase-independent cell death may occur through mitochondrial release of apoptosis-inducing factor (AIF), which translocates to the nucleus and promotes large-scale DNA fragmentation and chromatin condensation [17,18]. These canonical and non-canonical death pathways provide a conceptual foundation for understanding how tumor cells evade cell death and how specific RCD modalities may be therapeutically exploited in prostate cancer.

Accumulating evidence indicates that dysregulation of RCD plays a critical role in prostate cancer initiation, progression, and therapeutic resistance [19]. Tumor cells often acquire the capacity to evade cell death, thereby sustaining survival under endocrine, chemotherapeutic, and metabolic stress [10,19]. Conversely, therapeutic activation of specific RCD pathways may restore treatment sensitivity and enhance antitumor efficacy [20].

To address these issues, this review examines RCD in prostate cancer from an immunometabolic and translational perspective. We focus on how androgen receptor signaling, metabolic reprogramming, oxidative stress, and therapeutic stress shape the susceptibility of prostate cancer cells to major RCD modalities, including autophagy-associated cell death, ferroptosis, necroptosis, pyroptosis, and cuproptosis. We further discuss how these death-related pathways interact with the tumor immune microenvironment and how they may inform the development of combination therapies, nanodrug delivery approaches, and biomarker-guided treatment strategies for advanced prostate cancer.

Relevant research was identified through targeted searches of PubMed and Web of Science. Search terms included combinations of “prostate cancer”, “castration-resistant prostate cancer”, “regulated cell death”, “ferroptosis”, “autophagy”, “necroptosis”, “pyroptosis”, “cuproptosis”, “androgen receptor”, “metabolic reprogramming”, “tumor immune microenvironment”, “therapeutic resistance”, “nanomedicine”, and “biomarker”. Priority was given to mechanistic, translational, and clinically relevant studies related to prostate cancer or RCD biology. Rather than providing a formal systematic review or meta-analysis, this article aims to synthesize current evidence into a conceptual framework linking RCD biology with treatment resistance, immune remodeling, and therapeutic vulnerability in prostate cancer.

## 2. Major Forms of Regulated Cell Death in Prostate Cancer

**Figure 1:**
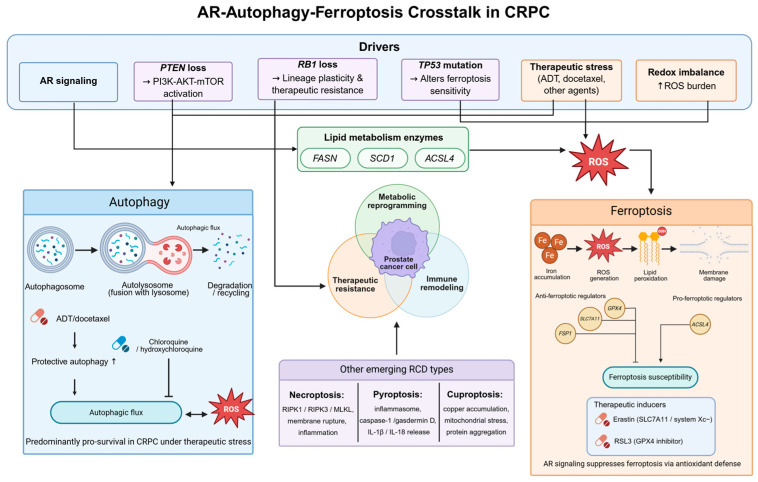
AR–autophagy–ferroptosis crosstalk in castration-resistant prostate cancer. Colors are used to distinguish functional modules rather than quantitative values: blue indicates AR/autophagy-related processes, green indicates lipid metabolic reprogramming, orange indicates redox/ferroptosis-related processes, and purple indicates CRPC-associated genomic alterations and other emerging RCD modalities. Black arrows indicate the direction of regulatory influence or signal flow, blunt-ended lines indicate inhibitory effects, and upward arrows indicate increased activity or burden. Created in BioRender. Wang, C. (2026) https://BioRender.com/7s14lca (accessed on 11 June 2026).

RCD pathways in prostate cancer do not operate as isolated events but instead form an interconnected regulatory network integrating metabolic signals, oxidative stress, and therapeutic stress responses (Figure 1) [14,15].

This schematic diagram illustrates how androgen receptor (AR) signaling, castration-resistant prostate cancer (CRPC)-associated genomic alterations, lipid metabolic reprogramming, oxidative stress, autophagic flux, and ferroptosis susceptibility are integrated into a shared regulatory network in advanced prostate cancer. AR signaling, *PTEN* loss-mediated PI3K–AKT–mTOR activation, *TP53* mutation, *RB1* loss, therapeutic stress, and redox imbalance act as upstream drivers that reshape cell death responses and adaptive survival programs. Lipid metabolism enzymes, including FASN, SCD1, and ACSL4, contribute to metabolic remodeling and influence reactive oxygen species (ROS) burden and ferroptosis vulnerability. In CRPC, autophagy predominantly functions as a stress-adaptive and pro-survival process during androgen deprivation therapy (ADT), docetaxel treatment, and other therapeutic pressures; however, excessive or dysregulated autophagic flux may contribute to cell death under severe stress. ROS may reciprocally interact with autophagic flux and also promote ferroptosis-related damage through iron-dependent lipid oxidation and membrane injury. Ferroptosis susceptibility is further controlled by anti-ferroptotic regulators, including SLC7A11, GPX4, and FSP1, and pro-ferroptotic lipid remodeling mediated by ACSL4 and PUFA-phospholipids. Ferroptosis inducers, including erastin and RSL3, may increase ferroptotic vulnerability, whereas AR signaling and antioxidant defense mechanisms may suppress ferroptosis execution.

Among the major RCD modalities implicated in prostate cancer, autophagy and ferroptosis have received particular attention because of their close association with metabolic reprogramming, redox homeostasis, and therapy resistance [13,21]. In addition, several emerging forms of RCD, including necroptosis, pyroptosis, and cuproptosis, have further expanded the mechanistic landscape of prostate cancer biology [19,22,23]. A better understanding of these pathways may reveal novel therapeutic vulnerabilities and provide a mechanistic basis for more effective treatment strategies. The major RCD modalities involved in prostate cancer are summarized in Table 1.

### 2.1. Autophagy: A Double-Edged Sword in Prostate Cancer

Autophagy is an evolutionarily conserved lysosome-dependent degradative pathway that sustains cellular homeostasis via recycling damaged organelles, misfolded proteins, and other intracellular components [24,25]. Through the formation of double-membrane autophagosomes and their subsequent fusion with lysosomes, intracellular cargo is degraded and reused for metabolic adaptation [25]. In cancer, autophagy is widely regarded as a context-dependent and double-edged process [26,27]. During early tumorigenesis, autophagy may exert tumor-suppressive effects by limiting oxidative stress, preserving genomic stability, and preventing the accumulation of damaged cellular constituents [26]. In established tumors, however, autophagy frequently promotes tumor survival by enabling adaptation to nutrient deprivation, hypoxia, and therapeutic stress [27].

Mechanistically, autophagy is closely regulated by the AMPK–mTOR signaling axis. Under metabolic stress or nutrient limitation, AMPK activation suppresses mTOR activity and promotes ULK1-dependent autophagosome initiation, thereby facilitating adaptive autophagy in tumor cells [28].

In prostate cancer, this adaptive function has been strongly linked to treatment resistance [29]. Androgen deprivation therapy can induce autophagy as a survival response to metabolic and hormonal stress, thereby enhancing tumor cell persistence [29,30]. Similarly, chemotherapeutic agents such as docetaxel may activate protective autophagy pathways, which in turn reduce treatment efficacy and contribute to drug resistance [31,32]. These observations suggest that autophagy constitutes an important mechanism by which prostate cancer cells tolerate hostile therapeutic microenvironments [29,30].

Consistent with this view, pharmacological inhibition of autophagy has been shown to enhance the sensitivity of prostate cancer cells to anticancer therapy [29,30]. Agents such as chloroquine and hydroxychloroquine inhibit lysosomal acidification and block autophagic flux, thereby sensitizing tumor cells to chemotherapy and targeted therapy [33,34]. Collectively, these findings indicate that autophagy is not only a critical adaptive mechanism in prostate cancer but also a potentially actionable therapeutic target, particularly in the context of combination strategies for advanced disease [29,30].

### 2.2. Ferroptosis: Metabolic Regulation and Androgen Signaling Crosstalk

Ferroptosis is a recently characterized form of RCD driven by iron-dependent lipid peroxidation [35,36]. Unlike apoptosis or classical necrosis, ferroptosis is characterized by the excessive accumulation of reactive oxygen species and lipid peroxides, ultimately leading to oxidative membrane damage and cell death [36,37]. Because prostate cancer is characterized by prominent metabolic reprogramming, especially in lipid metabolism and redox balance, ferroptosis is particularly relevant to its biological behavior [13,38].

In prostate cancer, androgen receptor signaling has been shown to influence several key ferroptosis-related regulators [39]. A central protective pathway is the cystine–glutathione–GPX4 axis. SLC7A11, a core component of system Xc−, promotes cystine uptake and supports glutathione synthesis, thereby maintaining intracellular antioxidant capacity and protecting tumor cells from ferroptotic damage [40,41]. Glutathione in turn serves as an essential cofactor for GPX4, which detoxifies lipid peroxides and suppresses ferroptotic cell death [42,43]. Increased SLC7A11 expression therefore enhances GPX4-dependent defense against ferroptosis [43]. In addition to this canonical pathway, ferroptosis suppressor protein 1 (FSP1) has emerged as an alternative defense mechanism that inhibits lipid peroxidation through coenzyme Q10 (CoQ10) reduction, further highlighting the complexity of ferroptosis regulation [44].

Another important determinant of ferroptosis sensitivity is ACSL4, which promotes the incorporation of polyunsaturated fatty acids into membrane phospholipids and thereby increases susceptibility to lipid peroxidation [45]. These findings underscore the close relationship between lipid metabolism and ferroptotic vulnerability in prostate cancer cells [13,46]. Given its role in redox homeostasis and metabolic adaptation, ferroptosis has emerged as a promising therapeutic target in advanced prostate cancer, particularly in castration-resistant disease [41,47]. Ferroptosis inducers such as erastin and RSL3 disrupt antioxidant defense systems by targeting SLC7A11 or GPX4, thereby promoting lethal lipid peroxidation [38,41]. Moreover, combining ferroptosis-inducing agents with androgen receptor-targeted therapies may represent a rational strategy for overcoming therapeutic resistance and improving treatment efficacy [39,48].

### 2.3. Other Emerging Regulated Cell Death Types

In addition to autophagy and ferroptosis, several emerging forms of RCD have recently attracted increasing attention in prostate cancer research, particularly necroptosis, pyroptosis, and cuproptosis [19,23,49]. Although the evidence supporting their functional roles in prostate cancer remains less extensive than that for autophagy and ferroptosis, these modalities may provide additional mechanistic insight into tumor progression, immune regulation, and therapeutic vulnerability.

Necroptosis is a regulated form of necrotic cell death mediated primarily by receptor-interacting protein kinases RIPK1 and RIPK3 together with the downstream effector MLKL [50]. Unlike apoptosis, necroptosis culminates in plasma membrane rupture and release of intracellular inflammatory mediators, thereby potentially influencing the tumor immune microenvironment and antitumor immunity [51,52]. In prostate cancer, this inflammatory nature makes necroptosis particularly interesting as a potential link between tumor cell death, stromal remodeling, and immune activation, although its context-specific consequences remain incompletely defined [51,52,53,54,55].

Pyroptosis is another inflammatory form of RCD mediated by members of the gasdermin family [56]. Activation of inflammasomes leads to caspase-1 activation and cleavage of gasdermin D, resulting in membrane pore formation and release of pro-inflammatory cytokines such as IL-1β and IL-18 [56,57]. Because of its pronounced immunostimulatory features, pyroptosis may be highly relevant to the crosstalk between tumor cell death and immune activation in prostate cancer [58]. However, whether pyroptosis predominantly enhances antitumor immunity or, under certain conditions, contributes to protumor inflammation in prostate cancer remains to be clarified [19,59].

Cuproptosis is a recently identified copper-dependent form of RCD characterized by mitochondrial protein aggregation, proteotoxic stress, and disruption of mitochondrial metabolism [23,60]. Although its role in prostate cancer remains less well characterized than that of autophagy and ferroptosis, emerging studies suggest that cuproptosis-related pathways may influence tumor progression, biochemical recurrence risk, immune landscape, and treatment responsiveness in prostate cancer [23,61,62,63]. Recent studies further indicate that copper-dependent death pathways may enhance sensitivity to androgen receptor-targeted therapy and docetaxel in preclinical models, raising the possibility that cuproptosis represents a novel metabolic vulnerability in advanced disease [64,65]. However, current evidence remains fragmented and is still dominated by exploratory mechanistic studies, bioinformatic analyses, and early preclinical therapeutic observations [23,66]. Therefore, substantial mechanistic and translational validation is still required before cuproptosis can be considered a clinically actionable target in prostate cancer [23,67].

Collectively, these emerging RCD modalities broaden the current understanding of prostate cancer biology and may provide new directions for mechanistic research and therapeutic development. Nevertheless, compared with autophagy and ferroptosis, their significance in prostate cancer remains less extensively characterized, and future studies are needed to clarify their biological context, immunological consequences, and therapeutic potential [23,68,69].

## 3. Crosstalk Between Regulated Cell Death and Tumor Immune Microenvironment

RCD not only determines tumor cell fate but also plays a crucial role in shaping the tumor immune microenvironment (TME) [49,70,71]. Increasing evidence suggests that dying tumor cells actively communicate with surrounding immune and stromal cells through the release of cytokines, metabolites, lipid mediators, and damage-associated molecular patterns (DAMPs) [49,72,73,74]. Depending on the specific mode of cell death and the biological context, these signals may either stimulate antitumor immunity or reinforce immune suppression [49,58,71]. Thus, RCD should be viewed not merely as a mechanism of tumor cell elimination, but also as a regulator of immune remodeling in prostate cancer [75,76,77].

**Figure 2:**
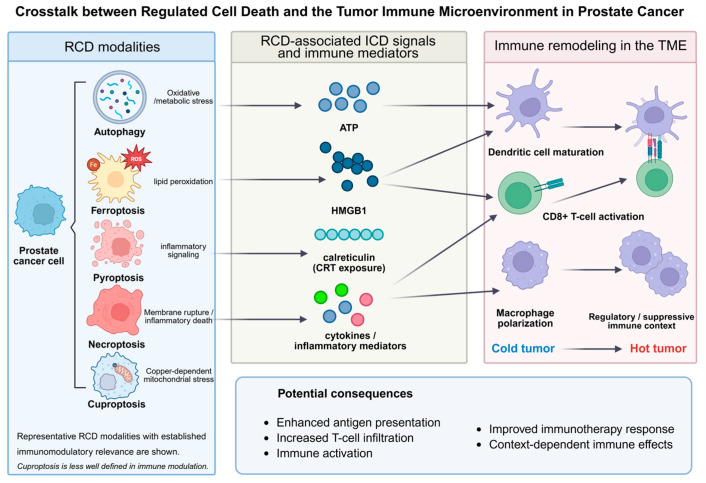
Crosstalk between regulated cell death and tumor immune microenvironment in prostate cancer. Colors are used to distinguish conceptual modules rather than quantitative values: blue indicates RCD modalities, the central light-green/gray module indicates RCD-associated ICD signals and immune mediators, pink/red indicates immune remodeling in the tumor immune microenvironment, and light blue indicates potential downstream consequences. Blue and red labels indicate immune-cold and immune-inflamed/hot tumor phenotypes, respectively. Arrows indicate the proposed direction of communication between RCD modalities, immune mediators, and immune-cell responses. Created in BioRender. Wang, C. (2026) https://BioRender.com/rh5f782 (accessed on 11 June 2026).

In prostate cancer, the TME is generally characterized by low immunogenicity, limited cytotoxic T-cell infiltration, and the presence of multiple immunosuppressive components, including tumor-associated macrophages, regulatory T cells, and myeloid-derived suppressor cells [75,78,79,80]. This immunologically “cold” phenotype is considered one of the major reasons why immune checkpoint blockade has shown only modest efficacy in unselected patients with advanced prostate cancer [79,81,82]. In this context, understanding how different forms of RCD influence immune activation, immune escape, and treatment responsiveness is of considerable translational importance. Elucidating the crosstalk between RCD and tumor immunity may therefore provide new opportunities for improving immunotherapy and developing rational combination strategies in prostate cancer [49,70,83,84]. As summarized in Figure 2, distinct forms of RCD can reshape the immune microenvironment in prostate cancer through immunogenic signaling, antigen presentation, immune cell recruitment, and context-dependent immune suppression.

Distinct forms of regulated cell death (RCD) can reshape the immune microenvironment in prostate cancer through the release of immunogenic signals and inflammatory mediators. Representative RCD modalities with established immunomodulatory relevance, including autophagy, ferroptosis, pyroptosis, and necroptosis, as well as the emerging modality cuproptosis, are shown. These death-associated processes generate key immune-related signals such as extracellular ATP, high-mobility group box 1 (HMGB1), calreticulin (CRT) exposure, and cytokines/inflammatory mediators, which influence dendritic cell maturation, CD8+ T-cell activation, macrophage polarization, and the balance between immune activation and immune suppression. Through these mechanisms, RCD may promote antigen presentation, increase T-cell infiltration, enhance immunotherapy responsiveness, and contribute to the conversion of an immunologically “cold” tumor into a more inflamed “hot” tumor. However, the immunological consequences of RCD are highly context-dependent, and some death-associated signals may also support regulatory or suppressive immune remodeling. Cuproptosis is included as an emerging RCD modality, although its immunomodulatory role in prostate cancer remains less well defined.

### 3.1. Immunogenic Consequences of Regulated Cell Death

Immunogenic cell death (ICD) describes a functional state in which dying tumor cells activate adaptive antitumor immune responses rather than representing a separate cell death pathway. In this process, specific forms of RCD are accompanied by the release or exposure of damage-associated molecular patterns (DAMPs), including extracellular ATP, high-mobility group box 1 (HMGB1), and CRT [85,86,87]. These signals function as endogenous danger cues that alert the immune system to the presence of dying tumor cells and promote immune recognition.

Mechanistically, calreticulin exposure on the tumor cell surface serves as an “eat-me” signal that promotes phagocytosis by dendritic cells. Extracellular ATP acts as a chemoattractant for antigen-presenting cells and supports inflammasome activation, whereas HMGB1 interacts with pattern-recognition receptors to enhance antigen processing and presentation [86,87]. Through these coordinated events, ICD promotes dendritic cell maturation and facilitates the priming and activation of tumor-specific cytotoxic T lymphocytes, ultimately leading to systemic antitumor immunity [86,88].

The concept of ICD is particularly relevant in prostate cancer because this tumor type is often considered poorly immunogenic and is typically characterized by limited immune cell infiltration and an immunosuppressive tumor microenvironment [79,81,89]. Promotion of ICD-associated immune signaling may therefore help transform an immunologically “cold” tumor into a more inflamed and therapeutically responsive phenotype [89,90,91]. This process may improve tumor antigen visibility, increase immune cell infiltration, and enhance the efficacy of immune checkpoint inhibitors or other immunomodulatory therapies [86,91].

Although ICD was initially described mainly in the context of chemotherapy- and radiotherapy-induced tumor cell death, accumulating evidence suggests that several RCD modalities may also generate immunogenic signals. For example, ferroptosis has been linked to the release of oxidized lipid mediators and inflammatory signals that may influence dendritic cell activation and T-cell recruitment [92,93,94]. Similarly, pyroptosis is characterized by membrane pore formation and release of pro-inflammatory cytokines such as IL-1β and IL-18, thereby potentially amplifying local immune responses [58,95,96]. These observations suggest that the immunogenic consequences of RCD may provide additional opportunities for therapeutic exploitation in prostate cancer [49,83].

### 3.2. Immune Modulation

Beyond directly inducing immunogenic responses, RCD can broadly remodel the tumor immune microenvironment by influencing the recruitment, activation, and functional polarization of immune cells [49,70,71]. Signals derived from dying tumor cells may shape the balance between immune activation and immune suppression, thereby affecting tumor progression and treatment response [70,97].

One important aspect of this process is the regulation of antigen-presenting cells. Death-associated signals can promote dendritic cell maturation and antigen cross-presentation, which are essential for effective T-cell priming [86,98,99]. In parallel, RCD-related mediators may influence the infiltration and activation of CD8+ cytotoxic T cells, the main effector cells responsible for tumor cell elimination [99,100]. However, these effects are highly context dependent, and under certain conditions cell death-associated signals may instead favor immune tolerance or dysfunctional T-cell responses [70,101,102].

RCD also affects myeloid cell populations within the TME, particularly macrophages [49,70]. Tumor-associated macrophages may adopt either a pro-inflammatory M1-like phenotype or an immunosuppressive M2-like phenotype depending on the cytokine and metabolic milieu generated during tumor cell death [77,103]. Some RCD pathways may support antitumor immunity by promoting inflammatory macrophage activation, whereas others may contribute to immune evasion by reinforcing M2 polarization and suppressive signaling [49,70]. In addition, RCD-associated factors may influence the accumulation of regulatory T cells and myeloid-derived suppressor cells, both of which are known to attenuate antitumor immune responses in prostate cancer [75].

Different forms of RCD may exert distinct immunomodulatory effects. Ferroptosis, for instance, can release lipid peroxidation products and inflammatory mediators that alter immune cell behavior, although its net impact may vary depending on the stage and intensity of ferroptotic stress [104,105]. Autophagy also has context-dependent immunological roles [106]. On the one hand, autophagy may facilitate antigen processing and support immune activation; on the other hand, it may help tumor cells survive under immune pressure and thereby contribute to immune escape [107]. These dual effects underscore the complexity of the relationship between RCD and tumor immunity [49].

From a translational perspective, therapeutic manipulation of RCD pathways may provide a strategy to reprogram the prostate cancer immune microenvironment and improve response to immunotherapy [49,84]. Combining RCD-inducing agents with immune checkpoint blockade, targeted therapy, radiotherapy, or nanomedicine-based approaches may simultaneously enhance tumor cell killing and promote immune activation [81,108,109,110]. Thus, a deeper understanding of RCD-mediated immune modulation may facilitate the development of more effective combination strategies for advanced prostate cancer [49,81].

## 4. Emerging Therapeutic Strategies Targeting Regulated Cell Death

**Figure 3:**
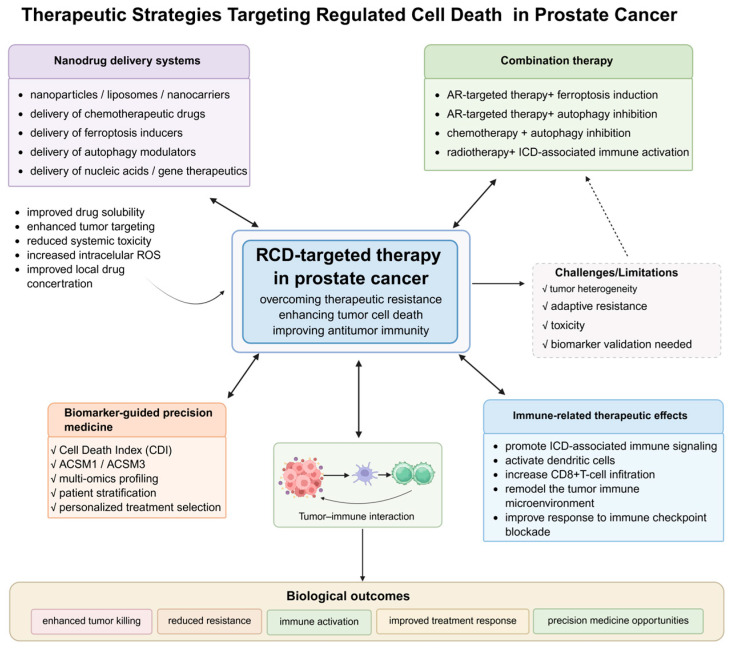
Therapeutic strategies targeting regulated cell death in prostate cancer. Colors are used to distinguish therapeutic modules rather than quantitative values: purple indicates nanodrug delivery systems, green indicates combination therapy, orange indicates biomarker-guided precision medicine, blue indicates immune-related therapeutic effects, light blue indicates the central RCD-targeted therapeutic framework, yellow indicates biological outcomes, and the gray dashed box indicates challenges and limitations. Solid arrows indicate conceptual links between therapeutic strategies, the central RCD-targeted framework, and downstream biological outcomes, whereas dashed arrows indicate factors that may limit or complicate therapeutic translation. Created in BioRender. Wang, C. (2026) https://BioRender.com/tvrneyw (accessed on 11 June 2026).

Targeting RCD pathways has emerged as a promising therapeutic strategy for prostate cancer, particularly in the setting of advanced disease and therapeutic resistance (Figure 3) [19,20]. Because tumor cells frequently evade conventional death programs during disease progression, restoring or selectively inducing RCD represents an attractive approach for improving tumor control [19,20,111]. Importantly, the therapeutic value of RCD modulation extends beyond direct tumor cell killing, as induction of specific death pathways may also alter tumor metabolism, increase oxidative stress, overcome resistance to endocrine or chemotherapeutic agents, and remodel the tumor immune microenvironment [10,19,49,112]. In recent years, advances in nanodrug delivery and rational combination therapies have substantially expanded the translational potential of RCD-based interventions in prostate cancer [49,109,113].

This schematic overview summarizes emerging therapeutic strategies aimed at modulating regulated cell death (RCD) pathways in prostate cancer. Major approaches include nanodrug delivery systems, combination therapeutic strategies, biomarker-guided precision medicine, and immune-related therapeutic effects. Nanotechnology-based platforms improve drug solubility, tumor targeting, and intracellular delivery of therapeutic agents, including ferroptosis inducers and autophagy modulators. Combination therapies integrating androgen receptor (AR)-targeted therapy, chemotherapy, radiotherapy, and RCD-inducing strategies may enhance treatment efficacy. Biomarker-guided precision medicine, including the Cell Death Index (CDI), ACSM1/ACSM3 expression, and multi-omics profiling, may facilitate patient stratification and personalized treatment selection. In addition, ICD-associated immune signaling may stimulate dendritic cell activation, promote CD8^+^ T-cell infiltration, and remodel the tumor immune microenvironment, thereby improving responses to immune checkpoint blockade. Despite these advances, challenges such as tumor heterogeneity, adaptive resistance, toxicity, and the need for biomarker validation remain. Ultimately, these strategies may lead to enhanced tumor killing, reduced therapeutic resistance, immune activation, and improved treatment outcomes.

### 4.1. Nanodrug Delivery Systems

Nanomedicine has emerged as an important platform for improving the delivery and efficacy of anticancer therapies [114,115,116,117,118]. Nanodrug delivery systems offer several advantages, including enhanced drug solubility, improved pharmacokinetics, better tumor accumulation, and reduced systemic toxicity [117,118,119]. These properties are especially valuable in prostate cancer, where therapeutic resistance and off-target adverse effects frequently limit treatment efficacy [114,115,120]. They are also highly relevant for RCD-targeting agents, many of which are constrained by poor stability, limited tumor selectivity, or dose-limiting toxicity [121].

Nanoparticle-based platforms can be engineered to deliver a broad range of therapeutic cargos, including conventional chemotherapeutic agents, nucleic acids, proteins, and small molecules that regulate RCD [119,122,123]. In the context of RCD-targeted therapy, nanocarriers may be used to deliver ferroptosis inducers, autophagy modulators, or nucleic acid-based regulators of cell death directly to tumor tissues, thereby improving local drug concentration and minimizing toxicity to normal cells [108,124,125].

Several recent studies provide more concrete examples of nanomedicine-enabled RCD modulation in prostate cancer. For instance, liposome-coated manganese sulfide/glucose oxidase nanoplatforms have been reported to enhance oxidative stress, promote lipid peroxidation, and induce ferroptosis-like tumor cell death in prostate cancer models [126]. Such systems illustrate that nanocarriers may function not only as passive delivery vehicles but also as active modulators of redox balance and ferroptotic vulnerability. In addition, iron-based nanomaterials and ferroptosis-inducing nanoparticle systems have been explored to improve tumor-selective delivery, intracellular retention, and local activity of ferroptosis-targeting agents. However, most of these strategies remain preclinical, and standardized prostate cancer-specific dosing or clinically translatable administration schedules are still lacking [12].

Another major advantage of nanodrug delivery systems is their ability to modulate the tumor microenvironment [109,117,127]. Certain nanomaterials can increase intracellular reactive oxygen species levels, disturb redox homeostasis, or alter iron metabolism, thereby sensitizing tumor cells to apoptosis or ferroptosis [128]. In addition, nanoparticle-based delivery may enhance the intracellular stability of labile compounds and facilitate co-delivery of multiple agents, which is particularly useful for combination therapies targeting distinct cell death pathways [129,130,131]. For example, nanocarriers can be designed to co-deliver ferroptosis inducers with chemotherapeutic agents, AR-targeted drugs, or immune modulators, thereby increasing local drug concentration while reducing systemic exposure [132]. Such multifunctional systems may be particularly valuable in advanced prostate cancer, where tumor heterogeneity and adaptive resistance frequently compromise the efficacy of single-agent treatment.

From a translational perspective, nanomedicine may also improve the therapeutic index of agents that would otherwise have limited clinical application due to poor solubility or systemic toxicity [119,133,134]. Nevertheless, further work is needed to define optimal drug loading, release kinetics, biodistribution, dosing schedules, long-term safety, and patient-selective biomarkers before RCD-oriented nanomedicine can be translated into routine prostate cancer treatment. Thus, nanodrug delivery systems represent a promising approach for maximizing the efficacy of RCD-based therapy in prostate cancer [114,133].

### 4.2. Combination Therapeutic Strategies

Given the molecular heterogeneity of prostate cancer and the adaptive capacity of tumor cells, single-agent therapy is often insufficient to achieve durable responses [135,136]. Combination therapeutic strategies that integrate RCD-targeting approaches with established anticancer treatments have therefore gained increasing attention [49,81,90]. The rationale for such approaches lies in their potential to simultaneously enhance tumor cell killing, overcome resistance mechanisms, and reshape the tumor microenvironment [49,90].

One important strategy is the combination of androgen receptor-targeted therapies with agents that modulate RCD [30,39]. Because persistent AR signaling remains a major driver of advanced prostate cancer, especially castration-resistant disease, combining AR inhibitors with ferroptosis inducers or autophagy inhibitors may enhance therapeutic efficacy and delay the development of resistance [21,39,47,137]. Preclinical studies have demonstrated that ferroptosis inducers such as erastin and RSL3 can suppress prostate cancer cell proliferation, impair tumor growth, and enhance oxidative damage by disrupting glutathione-dependent antioxidant defenses [138]. Notably, ferroptosis induction has shown potential synergistic activity when combined with AR-targeted therapies, including enzalutamide and abiraterone [138]. Mechanistically, AR inhibition may increase metabolic stress and alter redox homeostasis, thereby rendering prostate cancer cells more susceptible to lipid peroxidation and ferroptotic cell death. These observations support ferroptosis induction as a promising strategy for overcoming endocrine resistance in CRPC [48].

Tumor cells that survive endocrine therapy may become increasingly dependent on metabolic adaptation and antioxidant defense, thereby creating vulnerabilities that can be exploited through ferroptosis induction or autophagy blockade [30,39,47]. Autophagy inhibition provides another example of a rational combination strategy. In prostate cancer, AR-targeted therapy and chemotherapy may induce protective autophagy, allowing tumor cells to survive under therapeutic stress. Pharmacological autophagy inhibitors such as chloroquine (CQ) and hydroxychloroquine (HCQ) have therefore been investigated as adjuvant agents capable of restoring treatment sensitivity. For example, combined inhibition of androgen signaling and autophagy, such as abiraterone plus chloroquine, has been reported to enhance suppression of prostate cancer cell growth compared with either treatment alone [139,140]. Similarly, docetaxel-based regimens may induce adaptive autophagy, and autophagy blockade may increase tumor cell susceptibility to chemotherapy-induced death [141,142]. Combination approaches involving chemotherapy or radiotherapy may also benefit from RCD modulation [29,143]. Cytotoxic therapies often induce cellular stress but may fail to eliminate all tumor cells because of adaptive survival pathways [29,32]. In this setting, pharmacological targeting of autophagy or redox homeostasis may sensitize prostate cancer cells to conventional treatment and improve response durability [31,32,144]. Similarly, induction of inflammatory or immunogenic forms of cell death may increase the antitumor effects of radiotherapy by enhancing tumor antigen release and immune activation [145,146].

Another promising direction involves combining RCD-targeting agents with immunotherapy [90]. Because prostate cancer is generally considered an immunologically “cold” tumor, strategies that promote ICD-associated immune signaling or reprogram the tumor immune microenvironment may improve responsiveness to immune checkpoint blockade [79,81,147]. A representative conceptual strategy is the combination of AR inhibition, ferroptosis induction, and immune checkpoint blockade. Although such triple-combination strategies remain largely experimental, ferroptosis-associated release of DAMPs, inflammatory mediators, and lipid peroxidation products may enhance dendritic-cell activation and promote CD8+ T-cell recruitment. These effects could potentially improve the responsiveness of prostate tumors to immune checkpoint inhibitors and help convert immune-cold tumors into more inflamed and therapeutically responsive phenotypes. In this context, cancer vaccines and nanovaccine platforms may provide complementary strategies for amplifying RCD-associated antitumor immunity. By improving antigen delivery, adjuvant presentation, lymph-node targeting, and dendritic-cell activation, advanced nanovaccines may enhance tumor-specific T-cell priming and cooperate with ICD-associated immune signaling or immune checkpoint blockade. Although prostate cancer-specific nanovaccine evidence remains limited, these approaches provide a translationally relevant direction for converting RCD-induced immune signals into durable adaptive antitumor responses [148,149].

Ferroptosis, pyroptosis, and other inflammatory death modalities may amplify immune signaling, promote dendritic cell activation, and facilitate cytotoxic T-cell infiltration when appropriately incorporated into combination regimens [100,147,150]. Despite their promise, combination therapies targeting RCD also face several challenges, including drug scheduling, toxicity management, and patient selection [117,135,151]. In particular, optimal sequencing remains unclear: RCD-inducing agents may need to be administered before, concurrently with, or after endocrine therapy, chemotherapy, radiotherapy, or immunotherapy depending on the desired biological effect. Nevertheless, rationally designed combination regimens may ultimately provide a more effective therapeutic framework for advanced prostate cancer than monotherapy alone [135,152]. Their future success will likely depend on biomarker-guided patient selection, optimized treatment scheduling, and careful management of treatment-related toxicity [117].

### 4.3. Translational and Clinical Considerations

Despite substantial mechanistic progress, most RCD-targeted therapeutic strategies in prostate cancer remain at the preclinical stage [47,134]. Among the currently explored modalities, autophagy inhibition and ferroptosis induction have shown the strongest translational momentum, whereas necroptosis, pyroptosis, and cuproptosis are still supported mainly by mechanistic and exploratory evidence [12].

This translational gap reflects several unresolved issues, including insufficient biomarker-guided patient selection, limited validation in clinically relevant models, and the challenge of balancing efficacy with toxicity [117,153]. In addition, the context-dependent effects of RCD on tumor metabolism and the immune microenvironment indicate that therapeutic responses are unlikely to be uniform across all patients [90,112].

Accordingly, future efforts should focus on integrating mechanistic insights with clinically actionable biomarkers, improving model systems such as organoids and patient-derived platforms, and developing rational combination regimens that can be advanced toward clinical testing [117,153]. Representative studies on RCD-targeted therapeutic strategies in prostate cancer are summarized in Table 2.

## 5. Biomarkers and Precision Medicine

**Figure 4:**
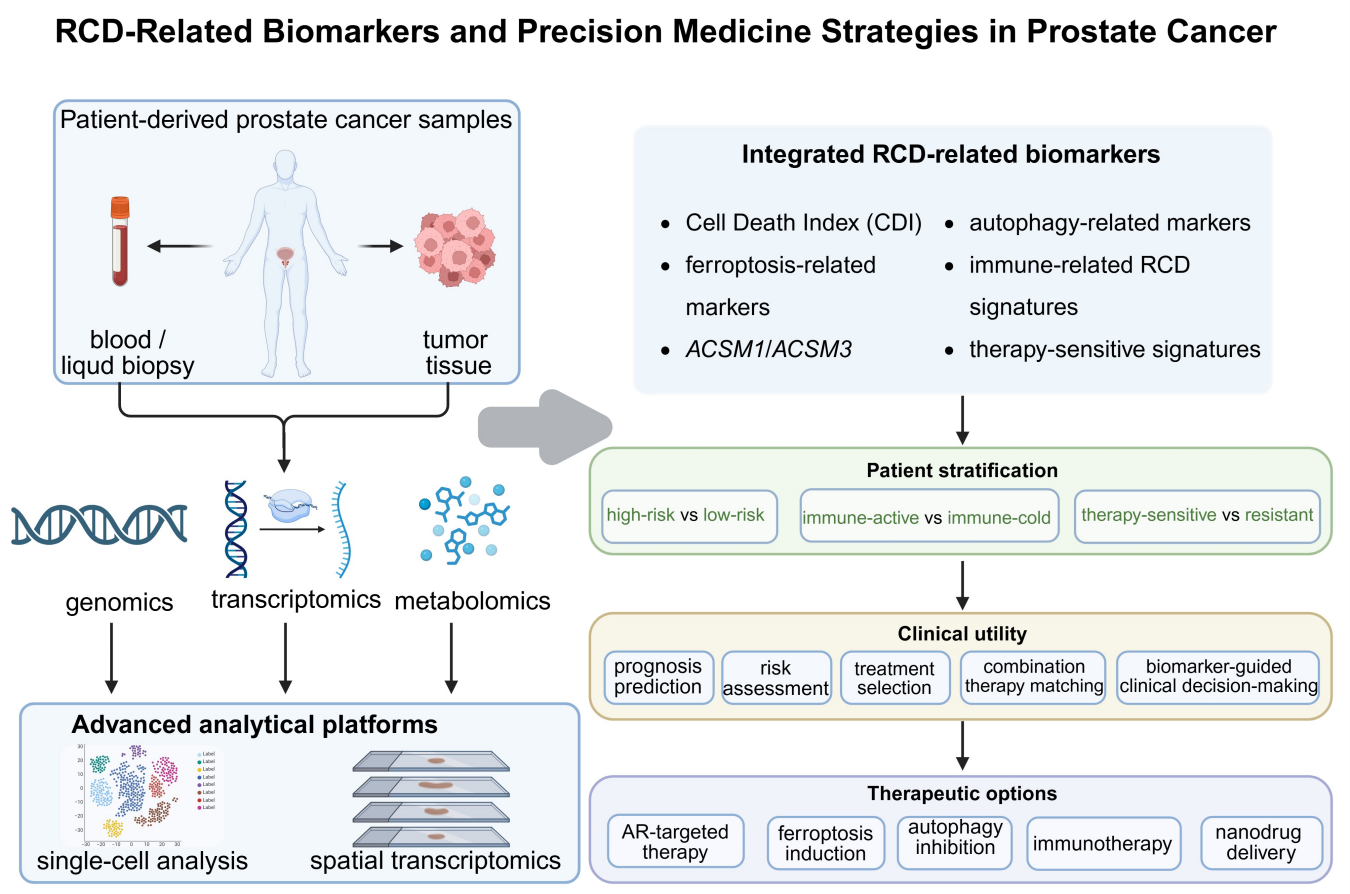
RCD-related biomarkers and precision medicine strategies in prostate cancer. Colors are used to distinguish conceptual modules rather than quantitative values: blue indicates patient-derived samples and analytical platforms, gray indicates integrated RCD-related biomarkers, green indicates patient stratification, yellow indicates clinical utility, and purple indicates therapeutic options. Arrows indicate the conceptual workflow from patient-derived samples and multi-omics profiling to biomarker identification, patient stratification, clinical decision-making, and therapeutic selection. Created in BioRender. Wang, C. (2026) https://BioRender.com/ui6hqgn (accessed on 14 June 2026).

Recent advances in genomic, transcriptomic, and metabolomic technologies have facilitated the identification of cell death-related biomarkers that may guide personalized therapy in prostate cancer [154,155,156,157,158]. As the biological heterogeneity of prostate cancer becomes increasingly recognized, there is growing interest in using molecular signatures to stratify patients according to prognosis, therapeutic vulnerability, and likelihood of treatment response [159,160,161,162,163]. In this context, biomarkers related to RCD may serve at least three complementary roles in precision oncology: prognostic stratification, prediction of therapeutic response, and treatment selection [159,160,163]. As summarized in Figure 4, patient-derived samples and multi-omics profiling may support the identification of integrated RCD-related biomarkers for patient stratification, clinical decision-making, and matched therapeutic strategies in prostate cancer [156].

This schematic overview illustrates how patient-derived prostate cancer samples and multi-omics profiling may support the identification and clinical application of regulated cell death (RCD)-related biomarkers in precision medicine. Tumor tissue and blood/liquid biopsy samples can be analyzed using genomic, transcriptomic, and metabolomic approaches, together with advanced analytical platforms such as single-cell analysis and spatial transcriptomics. These data may facilitate the identification of integrated RCD-related biomarkers, including composite signatures such as the Cell Death Index (CDI), individual mechanism-based markers such as *ACSM1*/*ACSM3*, and autophagy-, ferroptosis-, immune-, and therapy response-related signatures. Such biomarkers may contribute to patient stratification by distinguishing high-risk from low-risk disease, immune-active from immune-cold phenotypes, and therapy-sensitive from resistant tumors. In turn, these biomarker-guided frameworks may improve clinical utility by supporting prognosis prediction, risk assessment, treatment selection, combination therapy matching, and biomarker-guided clinical decision-making. Ultimately, RCD-related biomarkers may help guide matched therapeutic strategies in prostate cancer, including AR-targeted therapy, ferroptosis induction, autophagy inhibition, immunotherapy, and nanodrug delivery.

One representative example is the CDI, a composite molecular signature integrating genes involved in multiple cell death pathways [164]. Emerging studies suggest that CDI may have prognostic value in prostate cancer by reflecting the overall activity of death-related regulatory networks [164,165]. Patients with distinct CDI patterns may exhibit different clinical outcomes, molecular features, and immune microenvironment characteristics, indicating that integrated cell death-related signatures may help define biologically and clinically meaningful subgroups [161,165,166]. From a translational perspective, such signatures may also contribute to patient stratification by identifying tumors with distinct metabolic, immunological, or therapeutic vulnerabilities [157,162].

In addition to integrated signatures, individual genes associated with lipid metabolism and ferroptosis regulation have also attracted increasing attention [163,167]. For example, *ACSM1* and *ACSM3* have been implicated in prostate cancer progression and therapeutic response [167]. Because these molecules are closely linked to fatty acid metabolism and redox balance, they may influence the susceptibility of tumor cells to ferroptosis and other forms of metabolic stress [167,168]. Such mechanism-based biomarkers may therefore serve not only as prognostic indicators but also as potential therapeutic targets and predictors of treatment sensitivity [163,167,169].

Importantly, the clinical value of cell death-related biomarkers extends beyond prognosis alone [159,163]. These markers may help identify patients who are more likely to benefit from specific therapies targeting autophagy, ferroptosis, or other regulated death pathways [12,170]. Moreover, the integration of multi-omics data with clinicopathological variables may improve the predictive accuracy and clinical interpretability of biomarker models [154,159,170]. In this regard, advanced analytical approaches, including multi-omics integration, single-cell analysis, and spatial transcriptomics, may provide a more refined framework for biomarker discovery and treatment guidance in prostate cancer.

Nevertheless, several challenges remain before these biomarkers can be widely translated into clinical practice [159,171]. Many candidate biomarkers are still derived from retrospective datasets and require prospective validation in large, well-annotated patient cohorts [159,172]. In addition, standardized analytical pipelines, reproducible detection methods, and clinically feasible testing strategies are needed to ensure real-world applicability [171]. Despite these limitations, cell death-related biomarkers represent a promising direction for improving patient stratification, treatment selection, and precision medicine in prostate cancer [154,159].

## 6. Challenges and Future Perspectives

**Figure 5:**
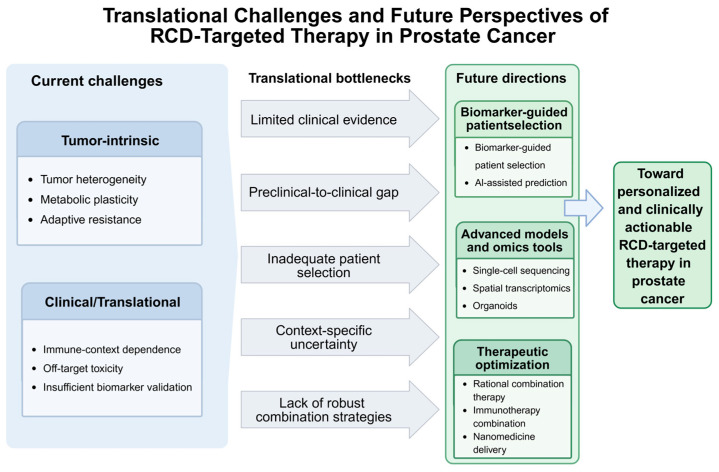
Translational challenges and future perspectives of RCD-targeted therapy in prostate cancer. Colors are used to distinguish conceptual categories rather than quantitative values, including translational challenges, key bottlenecks, future research directions, and clinically actionable therapeutic opportunities. Arrows indicate the conceptual progression from unresolved challenges to future strategies and toward more personalized and clinically applicable RCD-targeted therapy. Created in BioRender. Wang, C. (2026) https://BioRender.com/1s840lu (accessed on 11 June 2026).

Although significant progress has been made in understanding RCD in prostate cancer, several important challenges remain [173]. One of the major obstacles is tumor heterogeneity [174,175,176]. Prostate cancer exhibits substantial interpatient and intratumoral variability, meaning that the same cell death pathway may play different roles depending on disease stage, molecular subtype, treatment history, and microenvironmental context [174,177,178]. This complexity complicates the development of universally effective RCD-targeted therapies [173,179]. The major challenges, translational bottlenecks, and future directions of RCD-targeted therapy in prostate cancer are summarized in Figure 5 [153,180].

This schematic overview summarizes the major translational challenges, bottlenecks, and future directions associated with regulated cell death (RCD)-targeted therapy in prostate cancer. Current challenges include tumor-intrinsic factors, such as tumor heterogeneity, metabolic plasticity, and adaptive resistance, as well as clinical and translational constraints, including immune-context dependence, off-target toxicity, and insufficient biomarker validation. These limitations contribute to key translational bottlenecks, including limited clinical evidence, the preclinical-to-clinical gap, inadequate patient selection, context-specific uncertainty, and the lack of robust combination strategies. Future progress will likely depend on biomarker-guided patient selection, advanced models and omics tools, and therapeutic optimization. In particular, improved patient stratification, artificial intelligence-assisted prediction, single-cell sequencing, spatial transcriptomics, organoid-based models, rational combination therapy, immunotherapy-based combination therapy, and nanomedicine delivery may help accelerate the development of more personalized and clinically actionable RCD-targeted therapies for prostate cancer.

Another major challenge lies in adaptive resistance [173]. Even when a specific form of RCD is successfully induced, tumor cells may activate compensatory survival pathways, metabolic rewiring, or antioxidant defense mechanisms to evade lethal stress [112,136]. For example, ferroptosis induction may be offset by strengthened antioxidant defenses, whereas stress-induced autophagy may itself function as a survival pathway, thereby limiting long-term therapeutic efficacy [21,30,39,112]. Understanding these dynamic resistance mechanisms will be essential for designing more durable treatment strategies. [47,136].

The tumor immune microenvironment adds another layer of complexity [49,181,182]. Different forms of RCD may exert divergent immunological effects, ranging from immune activation to immune suppression [49,70]. As a result, the clinical outcome of targeting RCD pathways may depend not only on direct tumor cell killing but also on how these interventions reshape immune cell composition and function within the tumor microenvironment [49,181,182]. This highlights the importance of integrating tumor biology with immunological context in future therapeutic design [70,181,182,183].

From a translational perspective, the clinical development of RCD-targeted therapies also faces practical limitations, including insufficient biomarker-guided patient selection, potential off-target toxicity, and the lack of robust clinical validation for many experimental agents [159,183,184]. In addition, the gap between mechanistic discovery and clinical application remains substantial, emphasizing the need for therapeutic strategies that are not only biologically rational but also clinically feasible [162]. Future studies should therefore prioritize the identification of clinically actionable biomarkers, optimization of rational drug combinations, and validation of therapeutic approaches in preclinical models that better recapitulate human prostate cancer [171,185,186].

Emerging technologies may help address some of these limitations [162]. Single-cell sequencing, spatial transcriptomics, proteomics, and artificial intelligence-based analytical approaches are expected to provide deeper insights into the regulatory networks of RCD and their spatial and temporal heterogeneity within prostate tumors [170,187,188,189]. These technologies may also facilitate biomarker discovery, improve patient stratification, and support the identification of novel druggable vulnerabilities [156,187,189].

## 7. Conclusions

Overall, RCD represents an increasingly important biological and therapeutic framework in prostate cancer. By integrating tumor-intrinsic death pathways with metabolic reprogramming, immune remodeling, therapeutic resistance, and biomarker-guided treatment strategies, RCD-based research provides new opportunities for understanding and targeting advanced disease. In particular, ferroptosis, autophagy, pyroptosis, necroptosis, and cuproptosis may offer complementary vulnerabilities that can be exploited through rational combination therapy, immunotherapy, endocrine therapy, metabolic intervention, and nanomedicine-based delivery strategies [49,108,113,190]. However, the future clinical success of RCD-targeted therapy will likely depend on biomarker-guided patient selection, improved model systems, rational combination regimens, and continued translational validation [162,183,186]. A deeper mechanistic and clinically informed understanding of RCD may ultimately enable the development of more effective and personalized therapeutic strategies for prostate cancer [49,108,162].

## Figures and Tables

**Table 1:** Major regulated cell death modalities in prostate cancer: molecular features, immunological relevance, and therapeutic implications.

RCD Modality	Core Regulators/Molecular Features	Major Biological Consequences in Prostate Cancer	Immunological Relevance	Representative Therapeutic Strategies	Translational Relevance
Autophagy	ATG proteins, AMPK, mTOR, lysosomal degradation and recycling	Supports metabolic adaptation and stress tolerance; may suppress tumor initiation but promote progression and therapy resistance in advanced disease	Context-dependent; may affect antigen presentation, immune evasion, and response to therapy	Chloroquine, hydroxychloroquine, autophagy inhibition combined with AR-targeted therapy or chemotherapy	One of the most extensively studied RCD-related targets in prostate cancer, but therapeutic benefit likely depends on disease stage and molecular context
Ferroptosis	SLC7A11, GPX4, FSP1, ACSL4, iron-dependent lipid peroxidation	Induces oxidative membrane damage and restricts tumor survival; closely linked to metabolic vulnerability and treatment sensitivity	May enhance immunogenicity and influence the tumor immune microenvironment through lipid peroxidation-related inflammatory signaling	Erastin, RSL3, GPX4 inhibition, system Xc− targeting, ferroptosis-inducing nanodrug delivery	Among the most promising emerging therapeutic vulnerabilities, particularly in advanced and therapy-resistant prostate cancer
Necroptosis	RIPK1, RIPK3, MLKL, membrane rupture and inflammatory signaling	Causes inflammatory cell death and may reshape tumor progression, stromal interactions, and treatment response	Releases inflammatory mediators and may exert either pro-immunogenic or dysregulated inflammatory effects depending on context	Necroptosis induction, rational combination with radiotherapy or immunotherapy	Translational potential is emerging, but context-dependent biological effects and safety issues remain unresolved
Pyroptosis	Inflammasome, caspase-1, gasdermin family, IL-1β/IL-18 release	Triggers inflammatory tumor cell death and may alter progression, immune remodeling, and therapeutic response	Strongly associated with immunogenic and inflammatory signaling; may stimulate antitumor immunity but also promote protumor inflammation under specific conditions	Inflammasome activation, gasdermin-mediated strategies, combination with immunotherapy	Increasingly recognized as an immunologically relevant death modality, although therapeutic controllability remains limited
Cuproptosis	Copper accumulation, mitochondrial stress, lipoylated protein aggregation	Reflects a mitochondrial metabolic vulnerability and promotes proteotoxic stress in susceptible tumor cells	Immunological relevance is less well defined than that of other RCD modalities	Copper ionophores, modulation of copper homeostasis, mitochondria-targeted approaches	A newly emerging concept with mechanistic interest in prostate cancer, but currently supported mainly by exploratory studies

**Table 2:** Representative evidence, therapeutic implications, and current limitations of RCD-targeted strategies in prostate cancer.

RCD Modality	Representative Target/Strategy	Evidence Base in Prostate Cancer	Potential Therapeutic Context	Translational Implication	Current Limitations	References
Autophagy	Chloroquine or hydroxychloroquine-mediated inhibition of autophagic flux	Preclinical studies using prostate cancer cell lines and xenograft models	Combination with chemotherapy, AR-targeted therapy, or other stress-inducing treatments	Autophagy inhibition may suppress adaptive survival responses and enhance treatment sensitivity in selected contexts	Therapeutic benefit is context-dependent; optimal patient selection, timing, and combination partners remain unclear	[29,30,33,34]
Autophagy	Targeting stress-induced protective autophagy	Mechanistic studies linking ADT, chemotherapy, and metabolic stress to adaptive autophagy	Advanced or therapy-resistant prostate cancer	Supports autophagy blockade as a combination strategy rather than a universal monotherapy approach	Autophagy may also exert tumor-suppressive functions in early disease, raising concerns about disease-stage specificity	[29,30,31,32]
Ferroptosis	System Xc− inhibition and disruption of the cystine–glutathione–GPX4 axis	Preclinical studies showing ferroptosis induction and reduced tumor cell viability	CRPC, therapy-resistant disease, or tumors with redox and lipid metabolic vulnerabilities	Ferroptosis represents one of the most promising RCD-related vulnerabilities in prostate cancer	Clinical translation is limited by drug selectivity, systemic toxicity, and the lack of validated predictive biomarkers	[40,41,42,43,47]
Ferroptosis	GPX4 inhibition and lipid peroxidation induction	Cell line and animal model studies demonstrating oxidative membrane damage and ferroptotic cell death	Combination with AR-targeted therapy, chemotherapy, radiotherapy, or metabolic intervention	May overcome antioxidant defense and therapy resistance in metabolically adapted prostate cancer cells	Resistance may arise through compensatory antioxidant pathways such as FSP1-CoQ10 or altered lipid metabolism	[41,42,44,46]
Ferroptosis	Ferroptosis-inducing nanodrug delivery systems	Nanomedicine-based preclinical studies	Targeted delivery of ferroptosis inducers or combination cargos	Nanocarriers may improve tumor targeting, intracellular delivery, and therapeutic index	Most evidence remains preclinical; pharmacokinetics, safety, scalability, and clinical feasibility require further validation	[124,126,127,134]
Necroptosis	RIPK1/RIPK3/MLKL-related modulation	Early mechanistic and preclinical studies	Combination with radiotherapy, immunotherapy, or inflammatory tumor remodeling strategies	Necroptosis may enhance inflammatory signaling and contribute to antitumor immune activation under selected conditions	Biological consequences are highly context-dependent and may include protumor inflammation or tissue toxicity	[50,51,52,53,54]
Pyroptosis	Inflammasome activation and gasdermin-mediated tumor cell death	Mechanistic studies in prostate cancer models and immune-related exploratory analyses	Immune activation, immunotherapy sensitization, or combination with ICD-associated immune activation strategies	Pyroptosis may promote inflammatory tumor cell death and enhance immune visibility	Therapeutic controllability is limited; excessive inflammation and cytokine-related toxicity remain major concerns	[57,58,59,98,99]
Cuproptosis	Copper homeostasis modulation and mitochondrial proteotoxic stress	Emerging exploratory studies, including metabolic and bioinformatic analyses	Metabolically vulnerable tumors or combination with AR-targeted therapy and chemotherapy	Cuproptosis may represent a novel mitochondrial metabolic vulnerability	Evidence is still early and largely exploratory; prostate cancer-specific mechanisms require stronger experimental validation	[60,61,64,65]
Cuproptosis	Mitochondria-targeted copper stress	Early mechanistic observations	Advanced prostate cancer with mitochondrial metabolic dependence	Provides a potential non-apoptotic strategy for targeting therapy-resistant tumor cells	Clinical relevance, biomarkers of sensitivity, and safety profile remain insufficiently defined	[60,64,65]

## Data Availability

No new data were created or analyzed in this study. Data sharing is not applicable to this article.

## Abbreviations

The following abbreviations are used in this manuscript:

ACSL4	Acyl-CoA synthetase long-chain family member 4
ACSM1	Acyl-CoA synthetase medium-chain family member 1
ACSM3	Acyl-CoA synthetase medium-chain family member 3
ADT	Androgen deprivation therapy
AIF	Apoptosis-inducing factor
AKT	Protein kinase B
AMPK	AMP-activated protein kinase
AR	Androgen receptor
AR-V7	Androgen receptor splice variant 7
ATG	Autophagy-related
ATP	Adenosine triphosphate
CD8^+^	Cluster of differentiation 8-positive
CDI	Cell Death Index
CoQ10	Coenzyme Q10
CQ	Chloroquine
CRPC	Castration-resistant prostate cancer
CRT	Calreticulin
DAMPs	Damage-associated molecular patterns
FSP1	Ferroptosis suppressor protein 1
GPX4	Glutathione peroxidase 4
HCQ	Hydroxychloroquine
HMGB1	High-mobility group box 1
ICD	Immunogenic cell death
IL	Interleukin
IL-1β	Interleukin-1 beta
IL-18	Interleukin-18
MLKL	Mixed lineage kinase domain-like protein
mTOR	Mechanistic target of rapamycin
PCa	Prostate cancer
PI3K	Phosphoinositide 3-kinase
RCD	Regulated cell death
RIPK1	Receptor-interacting serine/threonine-protein kinase 1
RIPK3	Receptor-interacting serine/threonine-protein kinase 3
ROS	Reactive oxygen species
SLC7A11	Solute carrier family 7 member 11
TME	Tumor microenvironment
ULK1	Unc-51-like autophagy activating kinase 1
